# A repertoire of 124 potential autoantigens for autoimmune kidney diseases identified by dermatan sulfate affinity enrichment of kidney tissue proteins

**DOI:** 10.1371/journal.pone.0219018

**Published:** 2019-06-25

**Authors:** Wei Zhang, Jung-hyun Rho, Michael W. Roehrl, Michael H. Roehrl, Julia Y. Wang

**Affiliations:** 1 Department of Gastroenterology, Affiliated Hospital of Guizhou Medical University, Guizhou, China; 2 MP Biomedicals, Auckland, New Zealand; 3 Curandis, Scarsdale, New York, United States of America; 4 Department of Pathology and Human Oncology and Pathogenesis Program, Memorial Sloan Kettering Cancer Center, New York City, New York, United States of America; Instituto Nacional de Ciencias Medicas y Nutricion Salvador Zubiran, MEXICO

## Abstract

Autoantigens are the molecular targets in autoimmune diseases. They are a cohort of seemingly unrelated self-molecules present in different parts of the body, yet they can trigger a similar chain of autoimmune responses such as autoantibody production. We previously reported that dermatan sulfate (DS) can bind self-molecules of dying cells to stimulate autoreactive CD5+ B cells to produce autoantibodies. The formation of autoantigen-DS complexes converts the normally non-antigenic self-molecules to none-self antigens, and thus DS-affinity represents a common underlying biochemical property for autoantigens. This study sought to apply this property to identify potential autoantigens in the kidney. Total proteins were extracted from mouse kidney tissues and loaded onto DS-Sepharose resins. Proteins without affinity were washed off the resins, whereas those with increasing DS-affinity were eluted with step gradients of increasing salt strength. Fractions with strong and moderate DS-affinity were sequenced by mass spectrometry and yielded 25 and 99 proteins, respectively. An extensive literature search was conducted to validate whether these had been previously reported as autoantigens. Of the 124 proteins, 79 were reported autoantigens, and 19 out of 25 of the strong-DS-binding ones were well-known autoantigens. Moreover, these proteins largely fell into the two most common autoantibody categories in autoimmune kidney diseases, including 40 ANA (anti-nuclear autoantibodies) and 25 GBM (glomerular basement membrane) autoantigens. In summary, this study compiles a large repertoire of potential autoantigens for autoimmune kidney diseases. This autoantigen-ome sheds light on the molecular etiology of autoimmunity and further supports our hypothesis DS-autoantigen complexes as a unifying principle of autoantigenicity.

## Introduction

Autoimmune diseases are results of aberrant autoimmune responses. Immune defenses are normally generated against invading pathogens to protect the body. However, due to unclear mechanisms, the immune system sometimes deviates from its norm and produces undesirable autoimmune responses against the body itself. Autoimmune attacks can come from self-reactive cells and/or autoantibodies produced by autoreactive cells. Under normal circumstances, self-molecules in the body are non-antigenic, i.e., unable to trigger defensive immune reactions. It is puzzling how and why a self-molecule becomes an auto-antigenic trigger and/or target of autoimmune response. It is even more puzzling why, among the tens of thousands of molecules in the body, a cohort of only several hundred of seemingly unrelated molecules can trigger autoimmune reactions, e.g., production of autoantibodies.

In previous studies, we demonstrated that some molecules of dying cells have affinity for dermatan sulfate (DS), and that these molecules can form macromolecular complexes with DS to co-stimulate autoreactive CD5+ B cells to secrete autoantibodies [1]. Furthermore, we demonstrated that molecules with affinity for DS have a high propensity to be autoantigens (autoAgs) [2]. We thus proposed a unifying principle of autoantigenicity that explains how different molecules can become autoantigenic by means of a shared biochemical property. To gain further support, we have been testing whether autoantigens can be uncovered from specific tissues by enrichment with DS-affinity.

In this study, we applied the DS-affinity enrichment strategy to define the repertoire of possible autoantigens, i.e., the “autoantigen-ome”, in autoimmune kidney diseases. Although autoimmune attacks can happen in many parts of the body, they become especially serious if the kidneys are involved. Patients with autoimmune kidney diseases may develop glomerulonephritis. Glomeruli are made up of tiny blood vessels and help filter blood and remove excess fluids. When glomeruli are damaged, the kidneys stop working properly, which then leads to renal failure. Autoimmune renal diseases include lupus nephritis, Goodpasture syndrome or anti-GBM (glomerular basement membrane) disease, ANCA-associated vasculitis, and other rare diseases. They are largely defined by the kidney sub-location affected by the autoantibodies or immune cells. By identifying possible autoantigens in the kidney, we hope to gain a better understanding of the pathophysiology of these diseases.

## Materials and methods

### DS-Sepharose resin synthesis

DS-Sepharose resins were prepared by coupling dermatan sulfate (DS; Sigma-Aldrich) to EAH Sepharose 4B resins (GE Healthcare). Sepharose resins (20 mL) were washed with distilled water and 0.5 M NaCl and then mixed with 100 mg of DS dissolved in 10 mL of 0.1 M MES buffer (pH 5.0). N-ethyl-N-(3-dimethylaminopropyl) carbodiimide hydrochloride (Sigma-Aldrich) was added to a final concentration of 0.1 M. The reaction proceeded at 25°C for 24 hours with end-over-end rotation. After the first 60 minutes, the pH of the reaction mixture was readjusted to 5.0. After the coupling, the resins were washed three times, each time with a low pH buffer (0.1 M acetate, 0.5 M NaCl, pH 5.0) and a high pH buffer (0.1 M Tris, 0.5 M NaCl, pH 8.0). The washed DS-Sepharose resins were suspended in a 10 mM phosphate buffer (pH 7.4) and packed into a C16/20 column (GE Healthcare). The column was equilibrated with 10 mM phosphate buffer before use.

### Kidney protein extraction

Kidneys were obtained from 5-month-old BALB/cJ mice (Jackson Laboratory). A total of 20 mice were killed with CO_2_, their blood was removed through heart puncture, and their kidneys were collected immediately. The kidneys were cleaned by rinsing with phosphate buffered saline (PBS, pH 7.2) twice and then stored at 4°C for 1 hour, -20°C for 2 hours, and then -80°C until further processing. Thawed kidneys were cut to small pieces and pressed through a cell strainer (Fisher Scientific). To remove red blood cells (RBC), kidney tissue was mixed with 10 mL of RBC lysis buffer for 10 seconds. After centrifugation for 5 minutes, the supernatant was discarded. The tissue was mixed with 40 mL of RIPA lysis buffer (Sigma-Aldrich) and 4 tablets of protease inhibitor (complete protease inhibitor cocktail, Sigma-Aldrich). The tissue mixture was sonicated for 10 minutes, or until all tissue pieces appeared dissolved. The mixture was centrifuged at 13,300 rpm for 20 minutes, and the supernatant that contained all the soluble kidney proteins was collected. Protein concentration was measured by the RC DC protein assay (Bio-Rad).

### DS-affinity fractionation

Fractionation of kidney proteins was carried out by FPLC using a Biologic Duo-Flow System (Bio-Rad). Kidney proteins were loaded into the DS-Sepharose column in 10 mM phosphate buffer at a rate of 1 mL/min. The column was washed with 20 mL of 10 mM phosphate buffer and then 40 mL of 10 mM phosphate with 0.2 M NaCl to remove unbound proteins. Proteins bound to DS resins with moderate-to-strong affinity were eluted with a step-gradient of 0.4 M, 0.6 M, and 1.0 M NaCl in a 10 mM phosphate buffer, with each step being 40 mL. Elution was monitored by UV and conductivity detectors. All bound fractions were collected. Fractions were concentrated and desalted in Vivaspin centrifugal concentrators (MWCO 10 kDa, Sigma-Aldrich). Concentrated proteins were reconstituted in a 10 mM phosphate buffer for further analysis.

### Protein sequencing by mass spectrometry

Fractionated proteins with different affinity to DS were separated on 1D SDS PAGE in 4–12% NuPAGE Novex Bis-Tris gels (Invitrogen). Based on protein band intensity, the protein lane containing proteins eluting at 0.4 M NaCl was cut into three sections, containing top, middle, and bottom bands. The lanes containing proteins eluting at 0.6 M and 1.0 M NaCl were each cut into two sections, containing top and bottom bands, respectively. Gel sections were transferred into 1-mL tubes, cut into tiny pieces, dehydrated with acetonitrile, and then dried in a speed-vac. Proteins in gel pieces were then rehydrated in 50 mM NH_4_HCO_3_ and digested with 12.5 ng/μL modified sequencing-grade trypsin (Promega) at 4°C overnight.

Mass spectrometric sequencing was performed at the Taplin Biological Mass Spectrometry Facility (Harvard Medical School, Boston, USA). Tryptic peptides were separated on a nano-scale C18 HPLC capillary column and analyzed after electrospray ionization in an LTQ linear ion-trap mass spectrometer (Thermo Fisher Scientific). Peptide sequences and protein identities were assigned by matching protein or translated nucleotide databases with the measured fragmentation pattern using Sequest software. Peptides were required to be fully tryptic peptides with XCorr values of at least 1.5 (+1 ion), 1.5 (+2 ion), or 3.0 (+3 ion). All data were manually inspected. Only proteins with at least two peptide matches were considered confidently identified.

## Results and discussion

### Fractionation of kidney proteins by DS affinity

Proteins extracted from mouse kidneys were initially separated into fractions according to their strength of binding to DS. This was carried out by loading the kidney proteins onto DS-Sepharose columns to allow binding to take place. Proteins that did not bind to DS resins were washed off the column first with the loading buffer (10 mM phosphate, pH 7.4) and then with 0.2 M NaCl. Afterwards, proteins that remained bound to DS were sequentially eluted from the column with 0.4 M, 0.6 M, and 1.0 M NaCl in 10mM phosphate (pH 7.4). Elution was monitored for presence of proteins by 280 nm absorbance, and chromatographic fractions that contained proteins at each salt strength were pooled, desalted, and concentrated. Final protein content and size distribution of each pool were assessed with 1D SDS PAGE gels.

The majority of kidney proteins did not bind to DS, as most proteins flowed through or eluted at the 0.2 M NaCl washing steps. As the NaCl step gradient elution proceeded, the amount of proteins eluting at increasing ionic strength became smaller and smaller. The flow-through and non-DS-binding proteins were not further analyzed. Proteins eluting at 0.4 M, 0.6 M, and 1.0 M NaCl were sequenced by LC-MS/MS, yielding 124 proteins (Table 1).

**Table 1:** Kidney proteins with DS affinity.

# P	Protein ID	Gene	Protein	S	M	W	pI	Lit.
								
			**ANA (anti-nuclear autoantigens)**					
15	IPI00329998.3	H4	Histone H4	-		-	11.36	[3]
12	IPI00137852.5	H2afy	Core histone macro-H2A.1	-		-	9.81	[4]
10	IPI00153400.2	H2afj	Histone H2A.J	-		-	11.05	[4]
18	IPI00114642.4	Hist1h2bj	Histone H2B type 1-J	-		-	10.31	[5]
13	IPI00111957.3	Hist1h2ba	Histone H2B type 1-A	-		-	6.92	[5]
3	IPI00223713.5	Hist1h1c	Histone H1.2			-	11.00	[6]
3	IPI00404590.1	H1f0	Histone H1.0			-	10.90	[6]
17	IPI00317794.5	Ncl	Nucleolin			-	4.68	[7]
2	IPI00109764.2	Top1	DNA topoisomerase 1	-		-	9.35	[8]
4	IPI00123762.2	Rcc1	Regulator of chromosome condensation			-	8.34	[9]
4	IPI00111589.1	Cbx1	Chromobox protein homolog 1			-	4.85	[10]
4	IPI00118447.1	Pura	Transcriptional activator protein Pur-alph			-	6.06	
21	IPI00122011.2	Sf3b3	Splicing factor 3B subunit 3	-	-	-	5.13	[11]?
4	IPI00109860.3	Rbm8a	RNA-binding protein 8A	-		-	5.50	
2	IPI00222760.1	Prpf19	Pre-mRNA-processing factor 19	-			6.14	
8	IPI00121596.3	Prpf8	Pre-mRNA-processing-splicing factor 8 (U5 snRNP-specific protein)		-	-	8.95	[12]
2	IPI00284213.3	Prpf40a	Pre-mRNA-processing factor 40 homolog A (Renal carcinoma antigen NY-REN-6)			-	7.39	
7	IPI00128441.3	Hnrnpr	Heterogeneous nuclear ribonucleoportein R			-	8.23	[13]
5	IPI00620362.4	Hnrnpl	Heterogeneous nuclear ribonucleoprotein L			-	8.33	[14]
3	IPI00222208.2	Hnrnpul2	Heterogeneous nuclear ribonucleoprotein U-like protein 2			-	4.83	
2	IPI00406117.1	Syncrip	Heterogeneous nuclear ribonucleoprotein Q			-	8.68	
6	IPI00230541.3	Snrnp70	U1 small nuclear ribonucleoprotein 70 kD			-	9.94	[15]
2	IPI00119224.1	Snrpd3	Small nuclear ribonucleoprotein Sm D3			-	10.33	[16]
3	IPI00469260.3	Eftud2	U5 small nuclear ribonucleoprotein component 116 kDa			-	4.86	[17]
2	IPI00457815.3	Usp39	U4/U6.U5 tri-snRNP-associated protein 2			-	4.86	
2	IPI00128818.2	Dhx15	Pre-mRNA-splicing factor ATP-dependent RNA helicase DHX15			-	7.12	
2	IPI00284213.3	Prpf40a	Pre-mRNA-processing factor 40 homolog A (Huntington interacting protein A)			-	7.39	[18]
38	IPI00420807.3	Sfrs1	Serine/arginine-rich splicing factor 1			-	10.37	[19]?
6	IPI00153743.1	Sfrs7	Serine/arginine-rich splicing factor 7			-	11.89	
5	IPI00139364.1	Sfrs4	Serine/arginine-rich splicing factor 4			-	11.39	
2	IPI00121135.5	Sfrs2	Serine/arginine-rich splicing factor 2			-	11.86	
4	IPI00139259.1	Tra2b	Transformer-2 protein homolog beta (Serine/arginine-rich splicing factor 10)			-	11.25	
2	IPI00377298.2	Tra2a	Transformer-2 protein homolog alpha			-	11.28	
4	IPI00108271.1	Elavl1	ELAV-like protein 1 (Hu-antigen R)	-		-	9.12	[20]
3	IPI00109740.3	Mrpl2	39S ribosomal protein L2, mitochondria	-			11.07	[21]
3	IPI00555113.2	Rpl18	60S ribosomal protein L18	-			11.79	
2	IPI00222514.3	Mrps27	28S ribosomal protein S27, mitochondria	-			5.38	
2	IPI00119667.1	Eef1a2	Elongation factor 1-alpha 2			-	9.11	[22]?
3	IPI00762774.2	Eif3d	Eukaryotic translation initiation factor 3 subunit D			-	5.79	[23]?
4	IPI00134300.1	Ssb	Lupus La protein			-	6.68	[24]
								
			**Cytoskeleton / Basement Membrane**					
74	IPI00349520.5	Lrp2	Low-density lipoprotein receptor-related protein 2 (megalin)	-	-	-	4.94	[25]
18	IPI00123181.4	Myh9	Myosin-9	-		-	5.54	[26]
2	IPI00338604.5	Myh10	Myosin-10 (non-muscle myosin heavy chain B)			-	5.43	[26]
13	IPI00110827.1	Acta1	Actin, alpha skeletal muscle	-		-	5.23	[27]
72	IPI00118899.1	Actn4	Alpha-actinin-4			-	5.25	[28]
6	IPI00380436.1	Actn1	Alpha-actinin-1			-	5.23	[28]
7	IPI00126191.5	Lmnb2	Lamin-B2	-		-	5.40	[29]
3	IPI00400300.1	Lmna	Isoform C of Lamin-A/C	-		-	6.54	[30]
2	IPI00230394.5	Lmnb1	Lamin-B1			-	5.11	[31]
5	IPI00406213.4	Cspg4	High molecular weight melanoma-associated protein (melanoma chondroitin sulfate proteoglycan)		-		5.23	[32]
39	IPI00753793.2	Spna2	Spectrin alpha 2			-	5.20	[33]
26	IPI00121892.9	Spnb2	Spectrin beta 2			-	5.40	[33]
37	IPI00169916.1	Cltc	Clathrin heavy chain 1			-	5.48	
26	IPI00421223.3	Tpm4	Tropomyosin alpha-4 chain			-	4.65	[34]
26	IPI00123316.1	Tpm1	Tropomyosin alpha-1 chain			-	4.69	[35]
18	IPI00169707.2	Tpm3	Tropomyosin 3			-	4.68	[36]
12	IPI00109061.1	Tubb2b	Tubulin beta-2B chain			-	4.78	[37]
4	IPI00110753.1	Tuba1a	Tubulin alpha-1A chain			-	4.94	[37]
7	IPI00110588.4	Msn	Moesin			-	6.22	[38]
4	IPI00229509.2	Plec1	Plectin			-	5.74	[39]
4	IPI00227299.6	Vim	Vimentin			-	5.05	[40]
4	IPI00378698.6	Agrn	Agrin			-	5.91	[41]
2	IPI00395157.2	Epb41	Protein 4.1			-	5.43	[42]
2	IPI00125778.4	Tagln2	Transgelin-2			-	8.40	[22]?
2	IPI00462141.1	Gm5414	Type II keratin Kb14			-	6.88	[43]?
								
			**Cell Stress / Apoptosis**					
15	IPI00750217.1	Atg9a	Autophagy-related protein 9A	-			8.41	?
30	IPI00319992.1	Hspa5	78 kDa glucose-regulated protein (ER chaperone BiP)	-	-	-	5.07	[44]
5	IPI00133208.3	Hspa1l	Heat shock 70 kDa protein 1L	-		-	5.90	[45]
7	IPI00229080.7	Hsp90ab1	Heat shock protein HSP 90-beta	-		-	4.96	[46]
2	IPI00121788.1	Prdx1	Peroxiredoxin-1	-		-	8.26	[47]
11	IPI00118384.1	Ywhae	14-3-3 protein epsilon			-	4.63	[48]
6	IPI00230707.6	Ywhag	14-3-3 protein gamma			-	4.80	[48]
3	IPI00227392.5	Ywhah	14-3-3 protein eta			-	4.81	[49]
2	IPI00230682.6	Ywhab	14-3-3 protein beta/alpha			-	4.77	
4	IPI00118286.1	Sfn	14-3-3 protein sigma (Epithelial cell marker protein 1)			-	4.72	
9	IPI00468203.3	Anxa2	Annexin A2			-	7.55	[50]
2	IPI00129577.1	Aifm1	Apoptosis-inducing factor 1, mitochondrial			-	9.23	[51]
								
			**Miscellaneous**					
11	IPI00134809.2	Dlst	Dihydrolipoyllysine-residue succinyltransferase component of 2-oxoglutarate dehydrogenase complex	-		-	9.10	[52]
5	IPI00153660.4	Dlat	Dihydrolipoyllysine-residue acetyltransferase component of pyruvate dehydrogenase complex (70kD mitochondrial autoantigen of primary biliary cirrhosis)			-	8.81	[53]
4	IPI00130535.1	Dbt	Lipoamide acyltransferase component of branched-chain alpha-keto acid dehydrogenase complex (E2)			-	8.78	[54]
2	IPI00331555.2	Bckdha	Branched-chain ajpha-keto acid dehydrogenase E1 alpha chain			-	8.14	[55]
4	IPI00110843.3	Agmat	Agmatinase, mitochondrial	-			8.07	?
10	IPI00311682.5	Atp1a1	Na(+)/K(+)-transporting ATPase subunit alpha-1		-	-	5.30	?
5	IPI00121550.3	Atp1b1	Na(+)/K(+)-transporting ATPase subunit beta-1			-	8.83	[56]
4	IPI00122048.2	Atp1a3	Na(+)/K(+)-transporting ATPase subunit alpha-3			-	5.26	
2	IPI00131424.3	Cpt2	Carnitine O-palmitoyltransferase 2, mitochondrial		-		8.59	
30	IPI00271951.5	Pdia4	Protein disulfide isomerase A4			-	5.16	
13	IPI00122815.3	P4hb	Protein disulfide-isomerase			-	4.77	[57]
7	IPI00222496.3	Pdia6	Protein disulfide isomerase A6			-	5.00	
17	IPI00123639.1	Calr	Calreticulin			-	4.33	[58]
13	IPI00129960.1	Cdh16	Cadherin-16 (kidney-specific cadherin)			-	4.53	[59]?
3	IPI00119618.1	Canx	Calnexin			-	4.49	[60]
12	IPI00153317.3	Aldh1l1	Cytosolic 10-formyltetrahydrofolate dehydrogenase			-	5.64	
8	IPI00115679.1	Ganab	Neutral alpha-glucosidase AB			-	5.67	[61]
7	IPI00109122.1	Psma8	Proteasome subunit alpha type-7-like			-	8.81	
6	IPI00331644.5	Psma3	Proteasome subunit alpha type-3			-	5.29	
4	IPI00277001.4	Psma4	Proteasome subunit alpha type-4			-	7.58	
4	IPI00131845.1	Psma6	Proteasome subunit alpha type-6			-	6.34	
3	IPI00131406.1	Psma7	Proteasome subunit alpha type-7			-	8.59	[62]
3	IPI00420745.7	Psma2	Proteasome subunit alpha type-2			-	6.91	
2	IPI00283862.6	Psma1	Proteasome subunit alpha type-1			-	6.00	[63]
2	IPI00122562.3	Psma5	Similar to zeta proteasome chain			-	4.74	
6	IPI00129512.3	Psmb4	Proteasome subunit beta type-4			-	5.45	
5	IPI00113845.1	Psmb1	Proteasome subunit beta type-1			-	7.67	
4	IPI00317902.3	Psmb5	Proteasome subunit beta type-5			-	6.52	
3	IPI00119239.2	Psmb6	Proteasome subunit beta type-6			-	4.97	
2	IPI00136483.1	Psmb7	Proteasome subunit beta type-7			-	8.14	
2	IPI00128945.1	Psmb2	Proteasome subunit beta type-2			-	6.52	
7	IPI00622235.5	Vcp	Transitional endoplasmic reticulum ATPase			-	5.14	[64]
4	IPI00229963.6	Serpinb5	Serpin B5			-	5.55	[65]
4	IPI00135686.2	Ppib	Peptidyl-prolyl cis-trans isomerase B (cyclophilin B)			-	9.56	[66]
4	IPI00621548.2	Por	NADPH-cytochrome P450 reductase			-	5.34	[67]
2	IPI00131176.1	Mt-Co2	Cytochrome c oxidase subunit 2			-	4.60	[68]
4	IPI00318550.5	Ilf2	Interleukin enhancer-binding factor 2 (nuclear factor of activated T-cell 45 kDa)			-	5.18	[69]
3	IPI00279218.1	Apeh	Acylamino-acid-releasing enzyme			-	5.36	
3	IPI00169862.1	Coq9	Ubiquinone biosynthesis protein COQ9, mitochondrial			-	5.60	
3	IPI00228883.2	Pdzk1	Na(+)/H(+) exchange regulatory cofactor NHE-RF3 (CFTR-associated protein 70 kD)			-	5.29	
3	IPI00318108.2	Acox3	Peroxisomal acyl-coenzyme A oxidase 3			-	6.69	
3	IPI00330523.1	Pcca	Propionyl-CoA carboxylase alpha chain, mitochondrial			-	6.83	
2	IPI00319509.5	Anpep	Aminopeptidase N (CD13, gp150)			-	5.62	[70]
2	IPI00111908.8	Cps1	Carbamoyl-phosphate synthase [ammonia], mitochondrial			-	6.48	
2	IPI00119202.1	S100a11	Protein S100-A11			-	5.28	
2	IPI00122522.1	Ggt1	Gamma-glutamyltransferase 1			-	6.69	
2	IPI00126861.3	Tgm2	Transglutaminase 2			-	4.98	[71]

**Columns left to right:** Number of peptides identified for each protein by mass spectrometry; protein ID; gene name; protein name; S, strong DS affinity (eluted with 1.0 M NaCl); M, medium DS affinity (eluted with 0.6 M NaCl); W, weak DS affinity (eluted with 0.4 M NaCl); isoelectric point (pI) calculated from the full protein sequence; literature references describing autoantibodies for a protein (question marks indicate that autoantibodies for the protein in general were described, but exact target protein isoforms were not specified)

Proteins eluting at 1.0 M NaCl ionic strength were considered as having strong-DS affinity, whereas those eluteing at 0.6 M and 0.4 M were considered as having moderate and weak DS-affinity, respectively. Theoretical isoelectric points of all proteins were calculated using the pI compute tool of the ExPASy portal of the Swiss Institute of Bioinformatics (web.expasy.org/compute_pi/). These proteins span a wide range of pI values, ranging from very basic to acidic. Despite the fact that DS is acidic, there is no clear correlation between DS affinity strength and calculated pI values of these proteins.

### Proteins with strong DS affinity

Proteins eluting off the DS-Sepharose column at 1.0 M NaCl were considered as having strong DS affinity. From the 1.0 M elution, 25 proteins were identified by MS sequencing (Table 1). Among these 25 proteins, 13 were DNA-/RNA-associated, 5 were related to cytoskeleton/extracellular matrix, 5 were related to cell stress and/or apoptosis, and 2 were miscellaneous. Literature searches revealed that at least 18 (72%) of these proteins have previously been identified as target autoantigens for autoantibodies.

Histones, the proteins that organize DNA into nucleosomes, are the most prominent autoantigens identified with strong-DS affinity. Particularly, H4, two isoforms of H2A, and two isoforms of H2B were found in the 1.0 M elution. Two isoforms of H1 were also identified, but in the weak DS affinity fraction. Top1 (DNA topoisomerase 1), an enzyme that controls and alters the topology of DNA during transcription, was also identified by strong DS affinity. All six of these DNA-binding proteins are known autoantigens (see references in Table 1). In particular, antibodies to histones and nucleosomes are central in the pathogenesis of lupus nephritis.

The RNA-associated proteins identified in the strong DS affinity fraction are RBM8a (RNA-binding protein 8A), Sf3b3 (splicing factor 3B subunit 3), Prpf19 (pre-mRNA processing factor 19), Rmpl2 (39S ribosomal protein L2), Rpl18 (60S ribosomal protein L18), Mrps27 (28S ribosomal protein S27), and ELAV-like protein 1 (HuR). Elval1, also known as Hu antigen R, binds to and stabilizes mRNAs. HuR autoantibodies are a serologic hallmark of paraneoplastic encephalomyelitis [20]. Ribosomal P proteins are classical autoantigens, and autoantibodies to various other ribosomal proteins (such as L2, L5, L7, L12, L23a, S13, and S19) are reported but the heterogeneity awaits further characterization [21]. The specific L18 and Mrps27 ribosomal proteins identified here have not been reported as autoantigens. Autoantigen Sf3b1 (splicing factor 3B subunit 1) has been reported [11], but Sf3b3 identified here has not been reported as an autoantigen. Although similar RNA-binding proteins have been reported as autoantigens, RBM8a and Prpf19 have not been described as autoantigens.

DLST (dihydrolipopyllysine-residue succinyltransferase) is the E2 component of the 2-oxoglutarate dehydrogenase complex. It is mainly located in mitochondria. However, a small fraction localizes to the nucleus and participates in histone succinylation [72]. The RNA-binding autoantigen HuR is reported to affect the transcript of DLST [73]. DLST has been reported as an autoantigen [52].

All five proteins in the basement membrane or extracellular matrix family, including myosin 9, actin, lamins A and B2, and Lrp2 are known autoantigens (Table 1). Lrp2 (low-density lipoprotein receptor-related protein 2, also known as megalin) is found in the 1.0, 0.6, and 0.4 M elutions, suggesting its abundance in kidney tissue. Lrp2 is a multi-ligand endocytic receptor found in different tissues, but primarily in absorptive cells such as in the kidney. Its membranous expression is mainly seen in renal tubules and the parathyroid gland. Autoantibodies to Lrp2 have been found in patients with rheumatoid arthritis, systemic lupus erythematosus, Behçet disease, systemic sclerosis, and osteoarthritis [74]. In particular relation to kidney diseases, anti-Lrp2 and Lrp2 have been found in patients with kidney anti-brush border disease [25]. Immune deposits of anti-Lrp2 antibodies have been detected in the tubular basement membrane of affected kidneys.

Three heat shock proteins, Hsp5, Hspa1l, and Hsp90ab1, were identified in the strong DS affinity fraction (Table 1). HSPs are a group of stress-induced proteins and are thought to act as scavengers to trap abnormal proteins and protect stressed cells. HSPs share conserved structural motives, and autoantibodies to HSPs are frequently found in various autoimmune diseases [44–46].

Prdx1 (peroxiredoxin-1) plays a role in cell protection against oxidative stress by detoxifying peroxides and acting as a sensor of H_2_O_2_-mediated signaling events. Prdx1 was identified in the strong DS affinity fraction and is a reported autoantigen [47].

Agmat (agmatinase, also named agmatine ureohydrolase) was seen in the strong DS affinity fraction. Agmat has its highest expression level in the cortex of the kidney. Immunochemical studies have revealed that Agmat is expressed in the tubular epithelial cells of a normal kidney, but its expression is altered in renal cell carcinoma [75]. A role of Agmat in autoimmunity has not yet been described.

Atg9a (autophagy-related protein 9A) showed strong DS affinity. Atg9a is involved in autophagy and cytoplasm-to-vacuole transport vesicle formation. Autophagy is an intracellular degradation pathway. Metabolic stress can trigger the rearrangement of the 14-3-3ζ interactome to favor interaction with the core autophagy regulator Atg9A, resulting in enhanced phagosome production [76]. A number of 14-3-3 proteins were found in the weak DS affinity fraction (Table 1). It was recently reported that annexin A2 is an autophagy modulator that regulates autophagosome formation by enabling appropriate ATG9A trafficking from endosomes to autophagosomes via actin [77]. Annexin A2 was identified in the weak DS affinity fraction, whereas actin was identified in the strong DS affinity fraction. Although 14-3-3, Anxa2, and actin have been reported as autoantigens, the role of Atg9a in autoimmunity is still unclear.

### Proteins with medium DS affinity

From the fractions eluting at 0.6 M NaCl from the DS-affinity columns, 7 proteins were identified by MS, but 3 of them had already been present in the 1.0 M fraction. Therefore, only 4 new proteins were found (Table 1). The 3 proteins found in both the 1.0 M and 0.6 M fractions were Hspa5, Sf3b3, and Lrp2. The 4 new proteins were Prpf8, Atp1a1, Cpt2, and HMW-MAA.

Atp1a1 (Na+/K+-transporting ATPase subunit alpha 1) was found in the medium DS affinity fraction, whereas its two other subunits (Atp1a3 and Atp1b1) were found in the weak DS affinity fraction (Table 1). Atp1a1 (also called sodium pump subunit alpha-1) is the catalytic component of the active enzyme, catalyzing hydrolysis of ATP coupled with the exchange of Na+ and K+ ions across the plasma membrane. Mutations in the Atp1a1 gene causes dominant Charcot-Marie-Tooth disease 2DD [78]. Although autoantibodies to Atp1a1 have not been reported, an autoantibody to the beta-1 subunit has been described [56].

Cpt2 (carnitine O-palmitoyltransferase 2) is involved in the fatty acid-oxidation pathway, which is part of lipid metabolism. C2 deficiency caused by mutations in the Cpt2 gene is an autosomal recessive disorder of mitochondrial long-chain fatty acid oxidation, characterized by recurrent myoglobinuria, muscle pain, stiffness, and rhabdomyolysis. Myoglobinuria can cause kidney failure and death.

HMW-MAA (high molecular weight melanoma-associated protein) is also known as the melanoma chondroitin sulfate proteoglycan. Its expression is restricted in normal tissues but found in a large percentage of melanomas, and it has been used as a target for the immunotherapy of melanoma. Autoantibodies to this autoantigen were found in melanoma patients [32]. HMW-MAA is also a marker of activated pericytes [79]. Pericytes are contractile cells that wrap around the endothelial cells that line the capillaries and venules throughout the body. A deficiency of pericytes in the central nervous system can cause the blood-brain barrier to break down. Radiolabeled mAb immunoreactivity studies showed that HMW-MAA is highly expressed in the kidney and liver [80]. Possible autoimmune responses to HMW-MAA expressed in kidney microvascular pericytes may provide an important clue to renal capillary damage.

### Proteins with weak DS affinity

From fractions eluting at 0.4 M NaCl, 116 proteins were initially identified. However, 21 of them had also been detected in fractions eluted with 0.6 M or 1.0 M NaCl. After excluding the redundancies, 95 new proteins were discovered in the 0.4 M elution, and are these are regarded as having weak DS affinity (Table 1).

Among these 95 proteins, 26 are attributed to the ANA family, 19 to the basement membrane/extracellular matrix family, 7 to the cell stress/apoptosis family, and the rest (43) are miscellaneous. Of the 95 proteins, 52 (55%) are known autoantigens based on specific autoantibodies reported in literature (see references in Table 1).

Miscellaneous proteins that were verified as autoantigens by literature search include protein disulfide isomerase, calreticulin, calnexin, transglutaminase 2, neutral alpha-glucosidase, proteasome protein, transitional endoplasmic reticulum ATPase, serpin B5, cytochrome P450 reductase, cytochrome c oxidase, branched-chain alpha-keto acid dehydrogenase, and aminopeptidase (see references in Table 1).

Of particular interest are the 14 proteasome proteins identified in the weak DS affinity fraction. TGM2 autoantibodies are widely used in the diagnostics of celiac disease. It has come to be appreciated that these autoantibodies, and also the TGM2-specific B cells, might play an active role in the pathogenesis of celiac disease. TGM2 catalyzes the cross-linking of proteins and the conjugation of polyamines to proteins. It is believed to play a role in the pathogenesis of several diseases, including celiac sprue, neurodegenerative diseases, and certain types of cancer.

A few proteins that play significant roles in diseases, but against which autoantibodies have not been reported, include Ggt1 (gamma-glutamyltransferase 1 or glutathione hydrolase 1 proenzyme) and CDH16 (cadherin 16). Ggt1 is expressed in tissues involved in adsorption and secretion, particularly in the kidney. Gamma-glutamyltransferase is found in the renal microvascular compartment [81]. It is speculated that Ggt1 may contribute to the etiology of diabetes. However, the role of Ggt1 in autoimmunity has not yet been established. CDH16 was identified in the fraction with weak DS affinity. It belongs to the cadherin superfamily and is expressed in the kidney and the thyroid gland. Although CDH16 has not been described as an autoantigen, autoantibodies to vascular endothelial cadherin are reported to be a marker of endothelial dysfunction in autoimmune diseases [59].

Among autoimmune diseases of the kidney, lupus nephritis is the most common. Systemic lupus erythematosus (SLE) is a frequent trigger of nephritis. About two-thirds of patients with lupus develop inflammation in the glomeruli. ANA, particular anti-nucleosome autoantibodies, are serological markers of SLE. Nucleosomes are highly organized functional subunits of chromatin, consisting of ~2 turns of dsDNA wrapped around an octamer of 2 molecules of H2A, H2B, H3, and H4. Nucleosomes are joined by linker DNA associated with H1. In our study, we have identified different isoforms of H4, H2A, H2B, and H1 (Table 1). Anti-nucleosome antibodies have been shown to be a prognostic marker for SLE with renal involvement.

Goodpasture syndrome, also known as anti-glomerular basement membrane (anti-GBM) disease, is a prototype of autoimmune disease [82]. It is a rare disease in which autoantibodies attack the kidneys and lungs, particularly the glomerular and/or pulmonary small vessels or capillaries. The patients develop specific autoantibodies against basement membrane antigens. These autoantibodies deposit in the basement membrane and form immune complexes that activate the classical complement pathway. Subsequently, neutrophil-dependent inflammation occurs resulting in glomerulonephritis and/or alveolitis. Ultimately, chronic kidney disease and kidney failure result. The GBM is an unusually thick extracellular matrix, consisting of laminin, collagen, heparan sulfate proteoglycan, and other components. Thus far, the only characterized GBM autoantigen is type IV collagen [83]. In our current study, we identified 22 proteins potentially associated with GBM, including lamin, actin, spectrin, tropomyosin, tubulin, vimentin, myosin, and agrin. Except for clathrin, all others (21/22) have been reported as autoantigens in various diseases (see references in Table 1). It is possible that the 22 proteins identified here will provide additional diagnostic markers for anti-GBM disease.

Anti-neutrophil cytoplasmic antibodies (ANCA)-associated vasculitis is another autoimmune disease where abnormal autoantibodies attack small blood vessels in different parts of the body, including the kidney. Myeloperoxidase (MPO) and proteinase 3 (PR3), two ANCA antigens, are pathogenic players in ANCA vasculitis and have diagnostic value in renal vasculitis. However, MPO and PR3 are produced by infiltrating neutrophils in inflammation, but not by the normal vascular components of the kidney, which explains why these two autoantigens were not identified from the kidney tissue in this study.

## Conclusion

This study used the DS (dermatan sulfate)-affinity enrichment strategy and identified 96 proteins that are either verified or yet-to-be-confirmed autoantigens from kidney tissue. In particular, among these autoantigens, 22 proteins belong to the ANA (anti-nuclear antibodies) family, and 22 are related to the GBM or extracellular matrix, with ANA and GMB being two of the most characteristic autoantigens in autoimmune kidney diseases. In addition, this study identified 12 potential autoantigens that are related to apoptosis and/or cell stress, supporting the notion that apoptotic cells are an origin of autoantigens. Overall, our results strongly support a hypothesis for a unifying autoantigenicity principle, i.e., that self-molecules with affinity to DS have a high propensity to become autoantigens [1, 2]. The binding of DS to self-molecules may convert singular self-molecules (that are intrinsically non-antigenic) to non-self-complexes (that become antigenic), thereby inducing an autoimmune response. Our study demonstrates that our thesis about an underlying common property of autoantigens can be applied to identify known and to discover new renal autoantigens. A unifying physicochemical principle of autoantigenicity may eventually lead to therapeutic innovation by understanding the molecular etiology of autoimmunity.
